# Five-Month Follow-Up Assessing Defecography and Urodynamics after Laparoscopic Nerve-Sparing Colorectal Resection for Endometriosis

**DOI:** 10.1155/2020/8830867

**Published:** 2020-08-28

**Authors:** Claudio Peixoto Crispi, Claudio Peixoto Crispi, Alice Cristina Coelho Brandão Salomão, Claudia Maria Vale Joaquim, Bruna Rafaela Santos de Oliveira, Marlon de Freitas Fonseca

**Affiliations:** ^1^Crispi Institute of Minimally Invasive Surgery, Barra da Tijuca, Rio de Janeiro, RJ, Brazil; ^2^Fonte Imagem Medicina Diagnóstica, Lagoa, Rio de Janeiro, RJ, Brazil; ^3^Department of Proctology, Hospital Federal de Ipanema, Ipanema, Rio de Janeiro, RJ, Brazil; ^4^Department of Women's Health, Fernandes Figueira National Institute for Women, Children and Youth Health, Oswaldo Cruz Foundation, Rio de Janeiro, RJ, Brazil

## Abstract

**Introduction:**

Large resections may be necessary in cytoreductive surgery for endometriosis, which present risk of urinary and bowel complications. *Presentation of Case*. A 29-year-old woman underwent multidisciplinary laparoscopy for endometriosis in a private practice setting for acyclic pelvic pain and cyclic abdominal distension with changes in bowel habits and frequent sensation of incomplete defecation. After surgery, urodynamics remained normal and bowel function improved subjectively and objectively per dynamic magnetic resonance defecography (DMRD). The five-month follow-up found improvements in pain scores, bowel function, and health-related quality of life (assessed by the full versions of the Short Form 36 and Endometriosis Health Profile 30 scales). *Discussion*. Animus may contribute to the bowel symptoms in women with endometriosis. DMRD provides additional objective parameters for comparing pre- and postoperative functions.

**Conclusion:**

A nerve-sparing segmental rectosigmoidectomy for endometriosis carefully executed by a multidisciplinary team can preserve the function of different pelvic organs.

## 1. Introduction

Endometriosis is a remarkably frequent condition that affects women's health-related quality of life (HRQoL) [1]. Besides pain, infertility, and sexual dysfunction, endometriosis is also associated with bowel and lower urinary tract dysfunctions [2–4]. Cytoreductive surgery is the current treatment of choice to improve health-related quality of life in cases in which improvement with medical management has been unsatisfactory [5, 6]. Large resections may be necessary when multiple deep infiltrating lesions occur. An experienced multidisciplinary team should perform the surgery because of the risk of urinary [7] and bowel [8] complications.

Functional disorders of the pelvic floor, such as defecatory dysfunction, represent a common health problem in women. The integrity of the pelvic floor can be compromised by chronic inflammatory conditions of the pelvis, endometriosis chief among them [9]. For women with symptomatic intestinal and parametrial endometriosis refractory to medical management, cytoreductive surgery is often the next therapeutic option. Before undertaking surgery—increasingly performed using minimally invasive techniques—our team recommends a thorough preoperative assessment of pelvic function. The two studies we recommend are urodynamics [2] and dynamic magnetic resonance defecography (DMRD)—a special type of MRI, in which sequential images are obtained at various stages of defecation [9]. Here, we report the case of a young woman who underwent laparoscopic nerve-sparing surgery for deep infiltrating endometriosis (DIE) in a private practice setting, which included segmental colorectal resection. After an uneventful surgery and postoperative course, this case report discusses the clinical improvement observed through five months of follow-up with emphasis on pelvic function.

## 2. Case Summary

This case report was approved by an institutional review board, the Research Ethics Committee (CAAE 88571718.8.0000.5269 IFF-FIOCRUZ). Written consent was obtained and is on file at our institution. To improve transparency and reporting quality, the SCARE Guideline (consensus-based surgical case report) was used as a checklist (http://www.scareguideline.com).

A 29-year-old primiparous nonsmoking Caucasian woman was referred to our institution. She related pelvic pain due to endometriosis without fertility problems. The patient's past medical history included an uncomplicated cesarean section two years prior to the referral. After that, regular progestin therapy did not lead to satisfactory pain relief. Dysmenorrhea had occurred since menarche at age 11. Although she had used oral hormonal contraceptives from ages 19 to 26, the pain never resolved, neither during contraceptive intervals nor with vaginal spotting. Occasional hospitalizations for parenteral analgesia had been necessary. The patient experienced a progressive worsening of her pain over the course of the past four years, during which the pelvic pain became acyclic and almost constant. Preoperatively, no urinary symptoms were reported. However, concerning bowel function, cyclic abdominal distension, and changes in bowel habits were common as well as a frequent sensation of incomplete defecation.

## 3. Preoperative Assessment of Pelvis

The multidisciplinary pre- and postoperative findings are summarized in Tables 1–3.

The diagnosis of endometriosis suspected by clinical evaluation can be confirmed by magnetic resonance imaging (MRI) or ultrasound. In this case, pelvic MRI was performed in a specialized center by an experienced radiologist (A.C.C.B.S.). The images revealed deep infiltrating endometriosis (DIE) with multicompartmental endometriotic involvement, including the vesicouterine pouch, uterosacral ligaments, rectosigmoid, and left parametrium. Serum CA-125 was 17.3 units/mL during regular progestin use. As bowel and lateral parametrial endometriosis constitute a more severe manifestation of endometriosis [10], the preoperative assessment included a detailed pelvic assessment.

**Figure 1:**
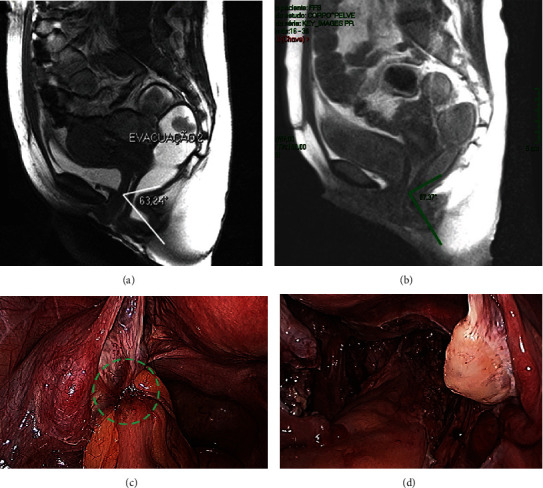
Sagittal dynamic magnetic resonance defecography: (a) assessment with Fast Imaging Employing Steady-State Acquisition (FIESTA) technique performed prior to surgery exhibiting paradoxical contraction of the puborectalis muscle during simulated defecation straining (anismus), a defecation anorectal angle of 63° (exerting stenosis); (b) assessment with single-shot fast spin echo sequence performed 3 months after nerve-sparing segmental colorectal resection identifying anismus, with a smaller reduction of the defecation anorectal angle of 87° and easier elimination of the rectal gel. Anorectal angle during defecation is normal when higher than at rest. The definitions and boundaries are according to Brandão and Ianez [9]. Panoramic laparoscopic view: (c) in the beginning of the surgery presenting intestinal endometriotic nodule adhered to the left uterine annex (dashed green circle); (d) after segmental colorectal resection with the rectosigmoid in anatomical position.

Preoperative rectosigmoidoscopy identified stenosis by angulation and external traction 15 cm from the anus, likely due to adhesions. Preoperative assessment of anorectal function by DMRD revealed a delay in the opening of the anal canal with intense anismus—paradoxical contraction of the puborectalis portion of the pelvic floor muscles—during the evacuation phase. This condition made it difficult to completely eliminate the study gel during the evacuation maneuvers (Figure 1(a)). The shift in the anorectal angle is considered normal within the ranges described by Brandão and Ianez [9], which are presented in Table 3.

Although the patient denied urinary symptoms, considerable involvement of the anterior and lateral compartments was identified by the MRI. Therefore, a more complete assessment of lower urinary tract anatomy and function was also performed preoperatively because of the possibility of subclinical abnormalities in micturition [2]. Cystoscopy revealed a normal bladder without endometriosis but detected thickening and retraction of the round ligaments bilaterally. Normal urodynamics were consistent with normal storage and voiding.

During the preoperative period, the HRQoL was assessed using the full versions of both the Short Form 36 and Endometriosis Health Profile 30 scales. The main endometriosis-related pain symptoms were quantified on a 0-10 visual analogue scale (VAS).

## 4. Surgery

An experienced multidisciplinary team led by a gynecologist (C.P.C.) performed the four-portal laparoscopic surgery under combined regional-general anesthesia. During exploration of the abdominal cavity, all lesions previously identified by physical examination and MRI were visually confirmed and resected. The harmonic scalpel-assisted cytoreductive surgery included the following: manual segmental rectosigmoidectomy (primary anastomosis), superficial resection (“shaving”) of external endometriotic lesions on the bladder, external adenomyomectomy, excision of the retrocervical region lesion with superior colpectomy, bilateral resection of the uterosacral ligaments, resection of the right round ligament, left parametrectomy, left oophoroplasty, right salpingoplasty, bilateral ureterolysis (dissection and identification), adhesiolysis, and fulguration of several endometrial peritoneal implants.

The surgery was uneventful, with a total duration of 190 minutes and negligible blood loss (<60 mL). All surgical specimens were assessed histologically. The presence of ectopic endometrial glandular epithelium was confirmed in all specimens. The laparoscopic panoramic views at the beginning of the surgery and after segmental colorectal resection are presented in Figures 1(c) and 1(d).

The patient was hospitalized for four days; there were no complications. As the intervention performed on the bladder was limited to resection of superficial external endometriotic lesions, the bladder catheter was removed within 24 hours. After the catheter was withdrawn, the postvoid residual urine volume was 90 mL. Prior to discharge, a 10.8 mg goserelin acetate implant was injected subcutaneously under the skin of the abdomen. Continuous combined anovulatory tablets (drospirenone/ethinyl estradiol) were initiated shortly thereafter. After discharge, the patient was seen monthly by the multidisciplinary team (gynecologist, proctologist, urologist, psychologist, and nutritionist) until the sixth month, when the patient was referred back to her regular gynecologist.

## 5. Postoperative Multidisciplinary Assessment

Even systematizing the entire laparoscopic surgery, including the delicate approach with the goal of maximum preservation of the pelvic nerves, functional complications may occur [11]. Thus, the great concern of this thorough multidisciplinary follow-up was to assess not only the reduction of pain and improvement of HRQoL (the standard outcome measures of this kind of endometriosis surgery) but also whether the surgery resulted in some damage to or functional worsening of the pelvic organs, particularly bowel and bladder functions.

Three months after the surgery, the patient no longer experienced cyclic abdominal distension or changes in bowel habits. She also reported an improvement in the frequency of defecation. Nevertheless, she still experienced a feeling of incomplete defecation, which for her was the most important intestinal preoperative complaint. Three months after surgery, rectosigmoidoscopy was normal and DMRD showed that the anorectal angles at rest and during the Valsalva maneuver were within the normal range, which suggests no remaining alterations in functional structure of the rectosigmoid after the segmental bowel resection and parametrectomy. The paradoxical contraction of the puborectalis muscle during simulated defecation straining (anismus) persisted, but the reduction of the anorectal angle was considerably less acute, enabling easier and more complete elimination of the rectal gel with less retention (Figure 1(b)). The urodynamic findings were normal five months after the surgery.

The variation of the anthropometric parameters during follow-up was minimal. Before surgery, at 163 cm height, the patient weighed 56 kg (body mass index = 21.3 kg/m^2^), the abdominal circumference was 80 cm, the lean body mass was 20.3 kg, and the body fat mass was 18.3 kg (bioelectrical impedance analysis). Her weight remained at 57 kg at 3 and 5 months after surgery (body mass index = 21.5 kg/m^2^).

## 6. Discussion

In summary, the five-month follow-up found improvements in bowel function, in HRQoL, and in most pain symptoms. The absence of postoperative (de novo) urinary dysfunctions supports the advantages of nerve-sparing techniques that have been heralded as a strategy for minimizing the risk of persistent urinary retention [7].

The sensation of incomplete evacuation (rectal tenesmus) is considered the most common symptom in patients with the diagnosis of rectosigmoid endometriosis [4]. In this case, this symptom still persisted after surgery, was less frequent, and was less intense. One explanation is that the anismus observed in both the pre- and postoperative DMRDs, which narrows the intestinal lumen, makes it difficult to completely eliminate the gel (and probably feces as well). Such anismus occurs in many patients who experience constipation and complain of a sensation of incomplete defecation, but in whom no significant underlying structural abnormality is encountered [9]. In the present case, although not infiltrating the pelvic floor muscles and thus not leading to structural alterations of the pelvic floor, the DIE did infiltrate the bowel and parametrium (a region that includes several pelvic nerves), among other sites. Moreover, the DMRD clearly demonstrated anismus even after segmental bowel resection and parametrectomy.

One can hypothesize that anismus could be due to pain during evacuation (dyschezia) or due to stenosis caused by adhesions or a transmural endometriotic nodule in the bowel wall. The patient, however, reported persistent “mild” anismus with a sensation of incomplete defecation, but no dyschezia. Moreover, the intestinal lumen appeared normal in the postoperative rectosigmoidoscopy.

More studies assessing the relationship between endometriosis and bowel function are needed. A PubMed search of the MEDLINE database was conducted on June 12, 2020, by two of the authors (C.P.C.Jr. and M.F.F.) using the combination of keywords “endometriosis AND defecography” and yielded just one recently published article. Sakala et al. [12] describe their technique using the dynamic mechanisms of MRI defecography to facilitate the diagnosis and mapping of pelvic endometriosis, particularly in cases of fibrotic or superficial lesions or in the rare malignant transformation of endometriomas. The authors do not address possible intestinal function impairment due to the presence of deep endometriotic lesions infiltrating the rectosigmoid, which is the focus of this case report.

Clinical evaluation of patients with pelvic floor dysfunction is difficult. Symptoms can be nonspecific, and the physical examination is frequently inaccurate. Endometriosis involving the bladder can disturb storage function, while endometriosis involving the parametrium impairs the voiding phase [2]. Therefore, we believe that the inclusion of urodynamic studies and defecography in the preoperative evaluation should be considered when major surgery for the treatment of endometriosis is planned. The documentation of possible preexisting pelvic disorders—regardless of whether they are associated with endometriosis or not—allows a more informed dialogue with the patient about the risks of undergoing a large surgery and the symptoms that might persist even after complete extirpation of the endometriotic lesions encountered.

In this case report, DMRD could detect problems in anorectal function both before and after laparoscopic nerve-sparing segmental colorectal resection for DIE. The hypothesis that anismus may contribute to the endometriosis-related bowel symptoms needs to be more thoroughly tested.

## 7. Conclusion

After assessing the changes in HRQoL scales, pain scores, and the anatomy and function of the urinary and bowel systems (subjectively and using urodynamics and defecography), we believe that a nerve-sparing segmental rectosigmoidectomy for DIE carefully executed by a multidisciplinary team can preserve the function of these pelvic organs. Although our operative experience suggests that the pelvic organ function can be improved, larger studies with a longer follow-up are needed to more completely assess late positive or negative consequences of this kind of surgery.

## Figures and Tables

**Table 1:** Health-related quality of life and pelvic pain assessment.

	Before surgery	3-month follow-up
Health-related quality of life: SF36 domains (0 represents the poorest health status)		
Physical functioning	100	100
Physical-role functioning	100	100
Bodily pain	51	72
General health perception	62	100
Vitality	70	85
Social-role functioning	75	100
Emotional-role functioning	100	100
Mental health	68	92
Health-related quality of life: EHP30 core instrument (0 represents the best health status)		
Pain	36.36	0
Control and powerlessness	70.83	0
Emotional well-being	79.17	12.50
Social support	56.25	0
Self-image	0	0
EHP30 supplementary optional modules (0 represents the best health status)		
Work	70	0
Relationship with child/children	25	0
Sexual relationship	0	10
Feelings about medical profession	0	0
Feelings about treatment	16.67	0
Feelings about infertility	50	0
Endometriosis-related pain symptoms		(0-10 scale)
Dysmenorrhea	10	0
Dyspareunia	5-10 (depending on the position)	0
Acyclic pelvic pain	10	0
Menstrual strangury	0	0
Menstrual dyschezia	0	0
Nonmenstrual dyschezia	0	0

Health-related quality of life scales (0-100): Short Form 36 (SF36; 0 represents the poorest health status) and Endometriosis Health Profile (EHP30; 0 represents the best health status). Endometriosis-related pain symptoms were quantified through a visual analogue scale (0-10).

**Table 2:** Bowel assessment.

	Before surgery	3-month follow-up
Rigid rectosigmoidoscopy		
Observations	Stenosis 15 cm from the anus	Normal up to 25 cm
Bowel function		
Cyclic changes in bowel movement	Tendency to diarrhea	No
Cyclic abdominal distension	Yes	No
Hematochezia	No	No
Feeling of incomplete evacuation (rectal tenesmus)	Sometimes	Milder and less frequent
Need to use of laxative to evacuate	No	No
Frequency	Once every 3 days	Once a day
Time to evacuate (min)	3	2
Anorectal function (DMRD)		
Anorectal angle at rest	93	109
Anorectal angle during squeeze (Valsalva)	69	72
Anorectal angle during defecation straining	63	87
Incomplete emptying	Yes	No
Slow emptying	Yes	No
Anismus	Yes	Yes, but less evident

Rigid rectosigmoidoscopy found no inflammatory disease. DMRD: dynamic magnetic resonance defecography. Anismus: paradoxical contraction of the puborectalis muscle during simulated defecation straining (this contraction reduces the anorectal angle, when there should be relaxation that increases the anorectal angle). Anorectal angle at rest: normal when between 70° and 134°. Anorectal angle during squeeze: normal when decreases more than 20°. Anorectal angle during defecation: normal when higher than at rest. The definitions and normal ranges are according to Brandão and Ianez [9].

**Table 3:** Urinary system assessment.

	Before surgery	5-month follow-up
Low urinary tract dysfunctions/symptoms		
Macroscopic hematuria	No	No
Renal calculi	No	No
Recurrent urinary tract infections	No	No
Straining to void	No	No
Feeling of incomplete emptying	No	No
Intermittent stream (intermittency)	No	No
Urgency	No	No
Urinary incontinence (leakage)	No	No
Strangury	No	No
Recurrent cystitis	No	No
Lumbar pain	No	No
Flank pain	No	No
Number of urinations per day	3	5
Nocturia	No	No
Self-reported urinary quality of life	Excellent	Excellent
Urodynamic measurements		
Bladder compliance (mL/cmH_2_O)	38	40
Maximum cystometric capacity (mL)	500	400
Opening pressure (cmH_2_O)	17	29
Maximum pressure (cmH_2_O)	23	45
Pressure at maximum flow (cmH_2_O)	21	31
Closing pressure (cmH_2_O)	25	-11
Maximum flow rate (mL/s)	23	20
Voided volume (mL)	500	395
Postvoid residual (mL)	0	5
Bladder outlet obstruction index	-25	-9
Bladder contractility index	136	131
Urodynamic observations		
Low bladder compliance	No	No
Detrusor underactivity	No	No
Abnormal bladder sensation	No	No
Detrusor overactivity	No	No
Abnormal residual urine	No	No
Bladder outlet obstruction	No	No
Maximum cystometric capacity < 300 mL	No	No
At least one abnormal finding	No	No

Self-reported urinary quality of life is a subjective question with an open answer. Recurrent urinary tract infections when 3 or more episodes per year (confirmed with urine culture). Low bladder compliance when <30 cmH_2_O. Detrusor underactivity when bladder contractility index (pressure at maximum flow + 5 × maximum flow rate) ≤100. Abnormal bladder sensation when the first desire to void occurs at cystometry < 80 or >200 mL. Detrusor overactivity when there are involuntary detrusor contractions during the filling phase. Abnormal residual urine when postvoid residual > 100 mL. Bladder outlet obstruction when bladder outlet obstruction index (pressure at maximum flow–2 × maximum flow rate) ≥40. The definitions and normal ranges are according to de Resende Júnior et al. [2].

## References

[1] de Freitas Fonseca M., Aragao L. C., Sessa F. V., Dutra de Resende J. A., Crispi C. P. (2018). Interrelationships among endometriosis-related pain symptoms and their effects on health-related quality of life: a sectional observational study. *Obstetrics & Gynecology Science*.

[2] de Resende Júnior J. A. D., Crispi C. P., Cardeman L., Buere R. T., Fonseca M. d. F. (2018). Urodynamic observations and lower urinary tract symptoms associated with endometriosis: a prospective cross-sectional observational study assessing women with deep infiltrating disease. *International Urogynecology Journal*.

[3] Surrey E. S., Soliman A. M., Johnson S. J., Davis M., Castelli-Haley J., Snabes M. C. (2018). Risk of developing comorbidities among women with endometriosis: a retrospective matched cohort study. *Journal of Women's Health (2002)*.

[4] Mabrouk M., Ferrini G., Montanari G. (2012). Does colorectal endometriosis alter intestinal functions? A prospective manometric and questionnaire-based study. *Fertility and Sterility*.

[5] Abrão M. S., Petraglia F., Falcone T., Keckstein J., Osuga Y., Chapron C. (2015). Deep endometriosis infiltrating the recto-sigmoid: critical factors to consider before management. *Human Reproduction Update*.

[6] Collinet P., Fritel X., Revel-Delhom C. (2018). Management of endometriosis CNGOF/HAS clinical practice guidelines short version. *Journal of Gynecology Obstetrics and Human Reproduction*.

[7] de Resende Júnior J. A. D., Cavalini L. T., Crispi C. P., de Freitas Fonseca M. (2017). Risk of urinary retention after nerve-sparing surgery for deep infiltrating endometriosis: a systematic review and meta-analysis. *Neurourology and Urodynamics*.

[8] Oliveira M. A. P., Pereira T. R. D., Gilbert A., Tulandi T., de Oliveira H. C., de Wilde R. L. (2016). Bowel complications in endometriosis surgery. *Best Practice & Research Clinical Obstetrics & Gynaecology*.

[9] Brandão A. C., Ianez P. (2013). MR imaging of the pelvic floor: defecography. *Magnetic Resonance Imaging Clinics of North America*.

[10] Mabrouk M., Raimondo D., Arena A. (2019). Parametrial endometriosis: the occult condition that makes the hard harder. *Journal of Minimally Invasive Gynecology*.

[11] Working group of ESGE, ESHRE, and WES, Keckstein J., Becker C. M. (2020). Recommendations for the surgical treatment of endometriosis. Part 2: deep endometriosis. *Human Reproduction Open*.

[12] Sakala M. D., Shampain K. L., Wasnik A. P. (2020). Advances in MR imaging of the female pelvis. *Magnetic Resonance Imaging Clinics of North America*.

